# Hippocampal vascularization patterns exert local and distant effects on brain structure but not vascular pathology in old age

**DOI:** 10.1093/braincomms/fcab127

**Published:** 2021-06-04

**Authors:** Niklas Vockert, Valentina Perosa, Gabriel Ziegler, Frank Schreiber, Anastasia Priester, Marco Spallazzi, Berta Garcia-Garcia, Merita Aruci, Hendrik Mattern, Aiden Haghikia, Emrah Düzel, Stefanie Schreiber, Anne Maass

**Affiliations:** 1 German Center for Neurodegenerative Diseases, 39120 Magdeburg, Germany; 2 Department of Neurology, Otto von Guericke University Magdeburg, 39120 Magdeburg, Germany; 3 J. Philip Kistler Stroke Research Center, Massachusetts General Hospital, Boston, MA 02114, USA; 4 Institute of Cognitive Neurology and Dementia Research, Otto-von-Guericke University, 39120 Magdeburg, Germany; 5 Department of Neuroradiology, University Hospital Heidelberg, 69120 Heidelberg, Germany; 6 Department of Medicine and Surgery, Unit of Neurology, Azienda Ospedaliero- Universitaria, 43126 Parma, Italy; 7 Biomedical Magnetic Resonance, Otto von Guericke University Magdeburg, 39120 Magdeburg, Germany; 8 Institute of Cognitive Neuroscience, University College London, London WC1N 3AZ, UK; 9 Center for Behavioral Brain Sciences (CBBS), 39106 Magdeburg, Germany

**Keywords:** vascularization, brain structure, cerebral small vessel disease, resistance, resilience

## Abstract

The hippocampus within the medial temporal lobe is highly vulnerable to age-related pathology such as vascular disease. We examined hippocampal vascularization patterns by harnessing the ultra-high resolution of 7 Tesla magnetic resonance angiography. Dual-supply hemispheres with a contribution of the anterior choroidal artery to hippocampal blood supply were distinguished from single-supply ones with a sole dependence on the posterior cerebral artery. A recent study indicated that a dual vascular supply is related to preserved cognition and structural hippocampal integrity in old age and vascular disease. Here, we examined the regional specificity of these structural benefits at the level of medial temporal lobe sub-regions and hemispheres. In a cross-sectional study with an older cohort of 17 patients with cerebral small vessel disease (70.7 ± 9.0 years, 35.5% female) and 27 controls (71.1 ± 8.2 years, 44.4% female), we demonstrate that differences in grey matter volumes related to the hippocampal vascularization pattern were specifically observed in the anterior hippocampus and entorhinal cortex. These regions were especially bigger in dual-supply hemispheres, but also seemed to benefit from a contralateral dual supply. We further show that total grey matter volumes were greater in people with at least one dual-supply hemisphere, indicating that the hippocampal vascularization pattern has more far-reaching structural implications beyond the medial temporal lobe. A mediation analysis identified total grey matter as a mediator of differences in global cognition. However, our analyses on multiple neuroimaging markers for cerebral small vessel disease did not reveal any evidence that an augmented hippocampal vascularization conveys resistance nor resilience against vascular pathology. We propose that an augmented hippocampal vascularization might contribute to maintaining structural integrity in the brain and preserving cognition despite age-related degeneration. As such, the binary hippocampal vascularization pattern could have major implications for brain structure and function in ageing and dementia independent of vascular pathology, while presenting a simple framework with potential applicability to the clinical setting.

## Teaser

Vockert et al. report that an augmented hippocampal vascular supply provides brain structural benefits at multiple levels, including medial temporal lobe substructure volumes and total grey matter volume, leading to improved cognition. Thus, the hippocampal vascular pattern could have major implications for ageing and dementia.

## Graphical Abstract

**Graphical Abstract:**
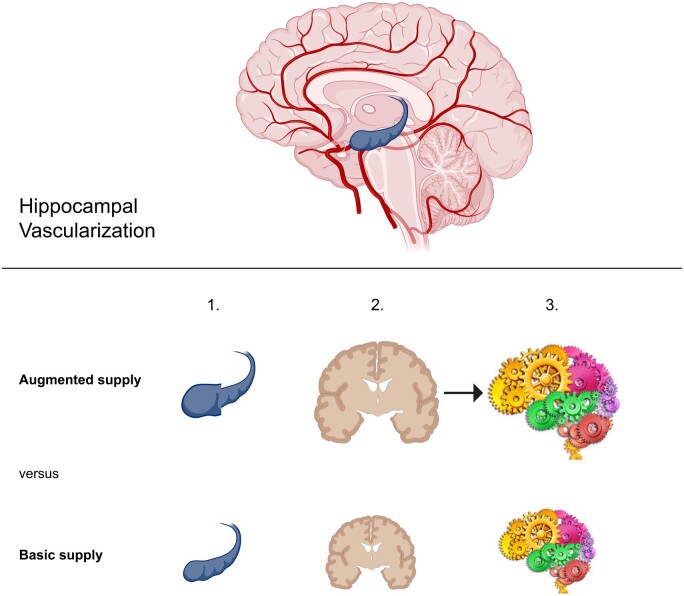

## Introduction

The hippocampus and its adjacent structures in the medial temporal lobe (MTL) are crucial for learning and memory.^1^ The MTL is a brain region especially affected by both normal and pathological ageing, matching the general observation of episodic memory decline over an adult lifetime.^2^ Inadequacy in terms of capillary density and afferent blood vessels makes the Cornu Ammonis in the hippocampus particularly vulnerable to shortcomings in blood supply.^3^

Despite the vast variability in hippocampal blood supply, previous post-mortem studies identified that all hippocampal arteries originate directly or indirectly from two arteries^4^^,^^5^: the posterior cerebral artery (PCA) and anterior choroidal artery (AChA). While hippocampal blood supply always depends on the PCA, the AChA contributes in ca. 40–45% of the examined hemispheres and primarily supplies the hippocampal head.^5^^,^^6^ Notably, the AChA, which originates from the internal carotid artery, belongs to the anterior circulation. In contrast, the PCA belongs to the posterior circulation in humans, a characteristic that distinguishes humans from other mammals.^7^ In brains where the PCA is the sole contributor to the hippocampal blood supply, the hippocampus is hugely dependent on the posterior circulation alone. The contribution of the AChA represents an additional involvement of the anterior circulation and could thus imply qualitative differences beyond the quantitative aspect of two vessels being more than one.

Sporadic cerebral small vessel disease (CSVD) is an umbrella term for alterations of the cortical and subcortical/deep cerebral microvasculature, which is very common in ageing, affecting the hippocampal vessels as well. It includes cerebral amyloid angiopathy (CAA) and deep perforator arteriopathy, also called hypertensive angiopathy.^8^ Age is a primary risk factor, such that almost all individuals aged 90 and about 80% of the 65-year olds exhibit clinical or radiological manifestations of CSVD.^9^ CSVD relates to cognitive decline in various domains, such as memory, and contributes to up to 45% of dementia cases.^10^^,^^11^

In the context of factors that modify pathology or its consequences, resistance and resilience refer to two important principles^12^: Resistance can be understood as avoiding pathology, i.e. exhibiting no major (or less than expected) pathological measures, like tau tangles in the case of Alzheimer’s disease or white matter hyperintensities (WMH) in CSVD. Resilience on the other hand means coping with pathology, i.e. remaining phenotypically (more) normal in spite of pathological changes. Arenaza-Urquijo and Vemuri^12^ introduce two definitions for resilience that are based on different viewpoints (see Box 1): (i) better brain structure or function (cognition) relatively to the amount of pathology or (ii) higher than expected pathology measures at a given level of cognitive performance or brain structure. Although originally defined in reference to Alzheimer’s disease, the concept can easily be transferred to other pathologies like CSVD. The pattern of hippocampal vascularization could be an important factor that relates to resistance or resilience in the context of vascular disease.

However, until recently, hippocampal arteries could only be studied post-mortem. With the use of a high-resolution 7T time-of-flight (ToF) magnetic resonance angiography sequence, Spallazzi et al.^6^ were able to visualize the small cerebral arteries in the MTL *in vivo* at an unprecedented resolution. Perosa et al.^13^ utilized this sequence for a binary classification of hippocampal vascularization in old adults with and without CSVD and discovered that a hippocampal vascularization pattern (HVP) with a combined hippocampal vascular supply of the PCA and AChA was linked to better cognition and overall hippocampal structural integrity using a voxel-based analysis.

Yet, the specific local and global associations between the HVP and structural brain integrity as well as vascular disease burden are still unknown. More specifically, it is unclear whether higher structural integrity related to a combined hippocampal vascular supply is only seen in the hippocampus or also extends to adjacent cortical regions such as the entorhinal cortex (ERC). This would have implications for ageing and Alzheimer's Disease, where the ERC shows the earliest neurodegeneration.^14^ Moreover, it is still unresolved whether structural differences or differences in vascular disease burden underlie the benefits in cognition seen in face of a combined hippocampal vascular supply. In addition, it remains elusive whether the HVP confers resilience or resistance to vascular disease. However, understanding their potential interplay could help to shape preventive and therapeutic strategies for CSVD.

Here, we aim to provide comprehensive insight into the relationship between the HVP and brain structure by investigating multiple unattended levels of detail, thereby advancing significantly on previous studies. First, we examine regional associations of the HVP with distinct MTL sub-regions. Second, we assess the specificity of these associations at the hemisphere level. Third, we examine the global effects of the HVP on total grey matter (GM) volumes and whether these explain the better cognitive performance observed in augmented-supply individuals. Furthermore, we assess markers of vascular disease burden including hippocampal microinfarcts and whole-brain white matter (WM) lesions as well as a semi-quantitative measure for overall CSVD severity in order to investigate the potential role of HVP in conferring resistance and resilience to vascular disease.

## Materials and methods

### Participants

This cross-sectional study was conducted using the same cohort as the study of Perosa et al.^13^ The cohort comprised 47 older adults (70.96 ± 8.22 years, 44% female), 20 of which showed neuroimaging markers of CSVD. The demographics of the participants can be found in Table 1. Exclusion criteria were depression as assessed by the Geriatric depression scale (GDS)^15^ and contraindications for scanning at 7T according to the recommendations of the German Ultrahigh Field Imaging (GUFI; https://mr-gufi.de Accessed 16 June 2021) network. All participants provided written informed consent according to the Declaration of Helsinki and were compensated for travel costs. The study was approved by the local Ethics Committee (93/17; 28/16).

**Table 1:** Demographics and vascular risk factors of the cohort with 20 CSVD patients and 27 controls

Variable	CSVD patients	Controls
Age (years)	70.7 *±* 8.5	71.1 *±* 8.2
Sex (% female)	35.0	44.4
Education (years)	14.3 *±* 4.1	16.0 *±* 2.5
MMSE	25.6 *±* 2.6	28.6 *±* 1.3
Arterial hypertension (%)	90.0	51.9
Diabetes mellitus (%)	25.0	11.1
Hyperlipidaemia (%)	60.0	44.4

Mean values ± SD are indicated.

The presence of CSVD was assessed on a 3T MRI by a specialized neurologist (S.S., 10 years of experience) according to the standards for reporting vascular changes on neuroimaging (STRIVE) criteria.^16^ CSVD neuroimaging markers comprised non-haemorrhagic [WMH, lacunes, enlarged perivascular spaces (PVS)] and haemorrhagic markers [cerebral microbleeds (CMB), cortical superficial siderosis]. Out of the 20 CSVD patients, one fulfilled the modified Boston criteria for a possible CAA and seven the criteria for probable CAA.^17^ The other 12 participants did not fulfil these criteria.

For the majority of CAA patients, CSF biomarkers for Alzheimer’s disease were available. The CSF amyloid β_1__*−*__42_ (Aβ_42_) and amyloid β_1__*−*__40_ (Aβ_40_) values of the six CAA cases that had CSF measures available were distributed reasonably along the values of a large CAA cohort, and can therefore be considered rather representative for (probable) CAA diagnosis (Supplementary Fig. 3). Based on the ATN classification scheme,^18^ an Alzheimer’s disease pathology profile could be established. Amyloid positivity (‘A’) was assessed with CSF Aβ_42_ levels, tau positivity (‘T’) with CSF phosphorylated tau (p-tau) levels and neurodegeneration (‘N’) with CSF total tau (t-tau) levels. Using locally established thresholds, only one CAA patient was classified as A^+^. However, none of the patients scored T^+^ or N^+^. One other (non-CAA) patient was also classified A^+^T^*−*^N^*−*^. Thus, two patients exhibited an Alzheimer’s pathological change along the Alzheimer’s continuum.^19^

All participants underwent a series of neuropsychological tests comprising the Mini-Mental State Examination (MMSE),^20^ Montreal Cognitive Assessment (MoCA),^21^ Alzheimer’s Disease Assessment Scale—Cognitive Subscale (ADAScog),^22^ Clinical Dementia Rating (CDR)^23^ and the German version of the California Verbal Learning Test II (CVLT-II).^24^

According to the CDR and MMSE, the CSVD cohort included 10 cognitively normal subjects (CDR = 0, MMSE *>* 26). Nine cases fulfilled the criteria for mild cognitive impairment (0 *<* CDR ≤ 1, 22 *<* MMSE ≤ 26), one participant fulfilled the criteria for mild dementia (CDR = 0.5, MMSE = 18). There was no participant with severe dementia (CDR *>* 1, MMSE ≤ 18). All control subjects were cognitively normal.

More information regarding the recruitment of the participants and the scanning protocol can be found in Perosa et al.^13^

### Image processing

#### Classification of the HVP

**Figure 1:**
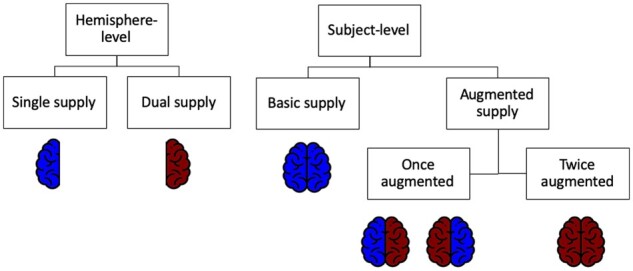
**Hippocampal vascularization patterns.** Terminology in regard to the dichotomous HVP on the level of a single hemisphere versus the level of an individual (whole brain). Red colour denotes a hemisphere with contribution of both the PCA and AChA to the hippocampal blood supply. Blue-coloured hemispheres denote an HVP without the involvement of the AChA.

The classification of the HVP occurred according to a binary scheme. For the purpose of clarity, the following terminology for the HVP at different levels is illustrated in Fig. 1. Hemispheres with a hippocampal blood supply encompassing both the AChA and the PCA are termed *dual-supply* hemispheres. In contrast, hemispheres without a contribution of the AChA to hippocampal vascularization are referred to as *single-supply* hemispheres. On the level of an individual, *basic supply* indicates two single-supply hemispheres, as opposed to an *augmented supply* that implies the presence of at least one hemisphere with dual supply. Thus, an augmented supply can further be subdivided into a *once augmented* and a *twice augmented supply*.

**Figure 2:**
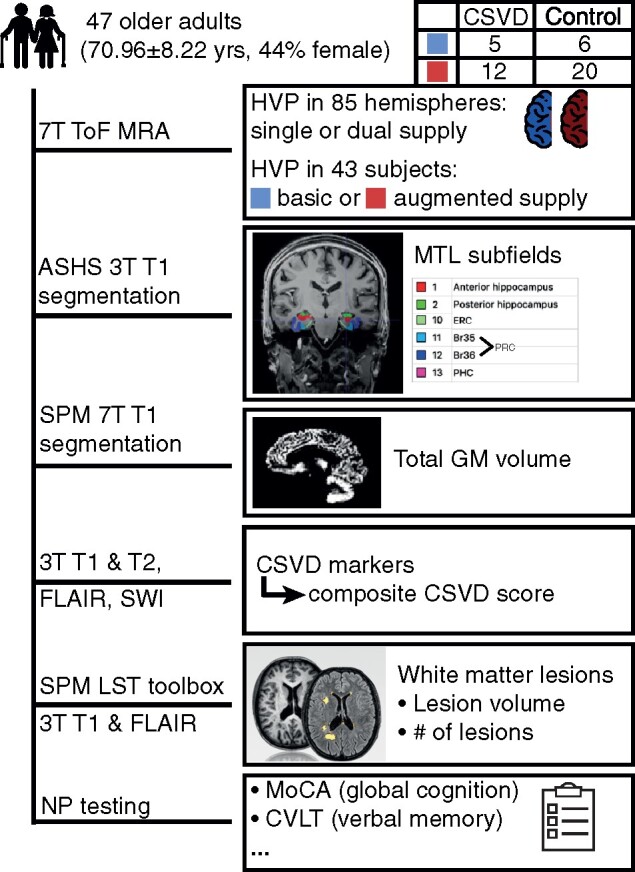
**Method overview. Illustrated are various steps prior to the statistical analysis of the data.** Please note that the 43 subjects or 85 hemispheres with classifiable hemispheres were used in subsequent analyses instead of the initial sample of 47 subjects.

In order to determine the HVP, the method described by Spallazzi et al.^6^ was adopted. For this purpose, the 7T T_1_-weighted images were bias-corrected with SPM12 (Statistical Parametric Mapping; Wellcome Trust Centre for Neuroimaging, London, UK) in MATLAB 2018a after conversion from DICOM to NIfTI. The bias-corrected images were automatically segmented with the subcortical segmentation pipeline of FreeSurfer 6.0. Visual inspection ensured the validity of the hippocampal segmentations. Using Advanced Normalization Tools (ANTs, http://stnava.github.io/ANTs/ Accessed 16 June 2021), the hippocampal masks were co-registered (affine transformation) to the 7T ToF magnetic resonance angiography images containing the angiographic information. An overlay of the ToF images with the hippocampal masks was visually inspected in MRIcron and MeVisLab to evaluate the contribution of the AChA to the hippocampal blood supply. If the uncal branch originating from the AChA was identified and seen penetrating the hippocampal region, the HVP of the hemisphere was classified as dual supply. However, in 9 of the 94 inspected brain hemispheres, the presence of the uncal branch originating from the AChA, and thus its participation to the hippocampal vascular supply, was doubtful (e.g. because the small vessels of the choroid plexus and its anastomoses could not be differentiated from the AChA or due to movement ar tefacts). Hence, 85 of the 94 hemispheres could be characterized in terms of their dichotomized HVP. This included 41 fully classified subjects and 3 partially classified subjects. Two of those three patients had a dual-supply hemisphere and thus could be counted towards the augmented-supply group, whereas the third had a single-supply hemisphere, making it impossible to characterize the subject on a subject level without the knowledge about the HVP in the other brain hemisphere. The joint distribution of the HVP to the clinical groups is displayed in Fig. 2.

The classification was performed by a trained medical student (A.P., 2 years of experience) and repeated several times in random order. Forty-two hemispheres of 21 subjects were additionally rated by a trained neurologist (V.P., 5 years of experience) to ensure inter-rater reliability (intraclass correlation coefficient = 0.83). The classification of the remaining hemispheres was verified by V.P. and consensus was reached when opinions diverged.

#### Analysis of brain structure

For brain structural analyses, the SPM12 segmentation routine was employed to segment the 7T T_1_ images into GM, WM and CSF. In order to account for the higher image inhomogeneities of 7T images, biasfwhm was set to 30 mm and samp (sampling distance) was set to 2. GM and WM volumes for every patient were calculated by summing up the voxel values of the corresponding individual tissue probability maps. Summing up all three corresponding tissue probability maps, total intracranial volumes (TIV) were obtained.

Moreover, volumes of MTL substructures were determined. An automatic segmentation was performed with the ASHS (Automatic Segmentation of Hippocampal Subfields) algorithm. Due to the lack of a high-resolution hippocampal T_2_-weighted image, the ASHS 3T Pennsylvania Memory Center atlas,^25^ which only requires a T_1_ image, was used. As the atlas was developed with 3T images, 3T (instead of the also available 7T) T_1_ images were utilized for MTL subfield segmentation. However, there was no disadvantage in terms of voxel size, as the 3T and 7T MPRAGE had the same resolution. The output of the segmentation (see Fig. 2) was the anterior hippocampus (aHC) (hippocampal head), posterior hippocampus (hippocampal body and tail), ERC, perirhinal cortex (PRC) divided into Brodmann areas 35 and 36 (BA35 and BA36) and parahippocampal cortex. The segmentations of the atlas were mostly based on the Harmonized Protocol)^25^ for manual hippocampal segmentation on T_1_-weighted MR scans.^26^ Since according to the Harmonized Protocol, the alveus and fimbria are included as part of the hippocampus,^27^ they were also included in the automatic segmentation.

**Figure 3:**
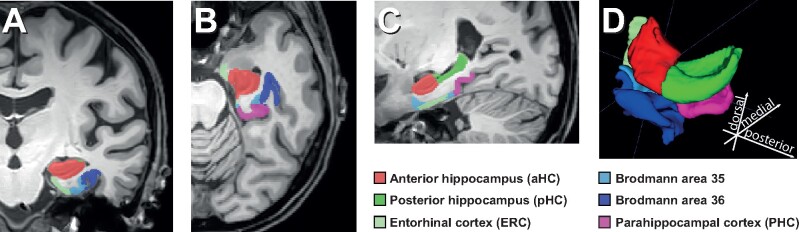
**ASHS segmentation. Manually curated ASHS segmentation of one participant.** Only one hemisphere is shown. (**A**) Coronal, (**B**) axial, (**C**) sagittal and (**D**) 3D view of the segmented MTL substructures.

The automatic segmentations were quality-controlled visually. Manual curation was done in absence of any subject information and focused on the correction of the three common errors mentioned in the supplementary of Xie et al.,^25^ namely: (i) under-segmentation of the lateral hippocampus border, (ii) segmentation of the choroid plexus as the hippocampus and (iii) over-segmentation of the MTL cortices. The correction of the segmentation borders was aided by the original segmentation protocol of the ASHS-T_2_-pipeline.^28^^,^^29^ Notably, some information like the exact border between the ERC and dura is not available in the T_1_ image alone.^25^ Furthermore, adjustments of the hippocampal border were aided by the Harmonized Protocol, which the automatic segmentations were also based on. Subsequently, the volumes were extracted from the regions of interest (ROIs). The following ROIs were considered for further analyses: aHC, ERC, PRC as sum of BA35 and BA36, posterior hippocampus and parahippocampal cortex. The segmentation of these substructures is shown exemplary for the left hemisphere of one participant in Fig. 3.

#### Assessment of CSVD severity

The lesion growth algorithm^30^ as implemented in the LST toolbox version 3.0.0 (www.statistical-modelling.de/lst.html) for SPM was utilized for quantification of WM lesions from the 3T T_2_ FLAIR and the 3T T_1_ images with default parameters (*κ  *=  0.3, threshold for binary lesion maps = 0.5). Total lesion volume as well as number of lesions were obtained and subsequently analysed as quantitative proxies for CSVD severity throughout the whole brain. Additionally, a semi-quantitative measure for overall CSVD severity was introduced based on two previously established scores.^31^^,^^32^ According to a review by Schreiber et al.,^8^ deep perforator arteriopathy has been particularly related to deep lacunes, deep/mixed CMB and PVS in the basal ganglia. CAA in contrast has been associated with strictly lobar CMB, cortical superficial siderosis, WMH and centrum semiovale PVS. As such, the score applied by Staals et al.^32^ is more specific to deep perforator arteriopathy. It assigns 1 point for one or more CMB in the cerebellum, brain stem, basal ganglia, WM or cortico-subcortical junction, 1 point for the presence of at least one lacune, and 1 point for Grades 2–4 PVS in the basal ganglia. In contrast, Charidimou et al.^31^ developed a score more specific to CAA. It accounts for lobar CMB (1 point for 2–4 CMB, 2 points for 5 or more), cortical superficial siderosis (1 point if focal, 2 if disseminated) and 1 point for Grade 3 or 4 centrum semiovale PVS. Both scores attributed 1 point to WMH when confluent deep WMH (Fazekas score 2 or 3) and/or irregular periventricular WMH extending into the deep WM (Fazekas score 3) were present. Combining the features from both scores, an overarching CSVD score for both subtypes was obtained, ranging from 0 to 9 points.

The number of hippocampal microinfarcts served as a local proxy for CSVD severity.

### Statistical analysis

All statistical analyses were performed in R version 3.6.1 if not stated otherwise. Most analyses were performed on the between-subject level rather than the level of single brain hemispheres. Therefore, subjects were split into two groups: Subjects in the basic-supply group had a single supply in both hemispheres, whereas the augmented-supply group contained individuals with a dual supply in at least one hemisphere (illustrated in Fig. 1). This split allowed to preserve more statistical power than a split into three groups, although a more detailed division would certainly be interesting to investigate.

In order to test whether an augmented supply is associated with local benefits in GM volume of the bilateral MTL structures compared to a basic supply, an MANCOVA was performed. To further test whether the HVP confers resilience against CSVD on a structural level (higher than expected volume at the same level of pathology), we additionally assessed the relation of the presence of CSVD to those measures and included an interaction term between the factors CSVD and HVP. Separate MANCOVAs were performed for anterior and posterior MTL structures. Anterior MTL structures included aHC, ERC and PRC (=BA35 + BA36), whereas the posterior hippocampus and parahippocampal cortex were examined as posterior structures. Age, sex and TIV served as covariates. Diabetes, hypertension and hyperlipidaemia showed no association with GM volumes and were thus not included as covariates. Univariate ANCOVAs with the same factors and covariates were performed as *posthoc* tests.

Differences in GM and WM volumes were assessed with ANCOVAs, again using the same factors and covariates. The same is true for the (M)ANCOVAs on CSVD measures. Permutation tests for the ANCOVAs were applied where deemed appropriate due to potential outliers in the small subgroups. Permutation tests were performed with the *permuco* package (method ‘manly’, 10 000 repetitions).^33^

Linear mixed effects (LMEs) models (implemented with the *lme4* package^34^) served as a tool to explore the directness of the hypothesized HVP effects on the basis of a single hemisphere on ipsilateral MTL structures. To account for the dependence of measurements for different hemispheres of the same individual, the LME modelled a different intercept for every subject. The covariates age and TIV were scaled (z normalized) to fall into similar ranges as the other variables. In the LME, both the HVP of the ipsilateral hemisphere and of the contralateral hemisphere were accounted for. Interaction terms between CSVD and the HVP were also included for both the ipsilateral HVP and the contralateral HVP. For example, the LME (full model) for the ERC looked the following:

$$\text{ERC}\;∼\;\text{age}+\text{sex}+\text{TIV}+\text{CSVDgroup}*{\text{HVP}}_{\text{ipsi}}+\text{CSVDgroup}*{\text{HVP}}_{\text{contra}}+\left(1\;|\;\text{id}\right)$$

Inference on the LME results was made with the use of confidence intervals. The use of *P-*values from *t* and *F* tests has been discouraged by Bates et al.,^34^ as sampling distributions of non-null estimates are not *t* distributed for finite sample sizes, nor are the corresponding null distributions of differences in scaled deviances *F* distributed. However, although confidence intervals and hypothesis tests are two sides of the same coin and confidence intervals are sufficient to guide inference, one might argue that *P*-values provide some complementary information.^35^^,^^36^ Hence, *P*-values calculated from likelihood ratio tests are additionally provided in the Supplementary material.

A mediation analysis was performed to test the hypothesis that total GM (TGM) volumes are responsible for the observed better global cognition in augmented-supply individuals, as assessed by the MoCA score. To compare memory and global cognition in this context, a composite memory score was constructed from different tasks of the CVLT. It was calculated as the sum of the z normalized scores for the sum of all five learning trials, the free short-delay task, the free long-delay task and a corrected hit rate (corrected for false alarm rate) from the recognition task.

The mediation analysis employed formal significance testing of the indirect path with a bootstrapping approach (50 000 iterations) as suggested by Preacher and Hayes.^37^^,^^38^ The *mediation* package supplied the required utility.^39^ Prior to testing of the indirect path, associations between the independent variable (HVP) and the mediator variables (TGM volumes or ROI GM volumes) as well as the dependent variables (cognition or verbal memory) were tested for significance. As the inclusion of TIV did not seem appropriate in every model, GM volumes were corrected for TIV after Raz et al.^40^ for every subject *i*:

(1) $$Vol_{cor_{i}}=Vol_{raw_{i}\;}-b(TIV_{i}-TIV_{\mathrm{mean}})$$

where *b* is the slope of the ROI volume regressed on TIV. Consequently, TIV was omitted as a covariate from the mediation models.

### Data availability

De-identified data are available from the corresponding author upon reasonable request subject to a material transfer agreement.

## Results

### HVP is associated with bigger anterior but not posterior MTL GM volumes

The relation between the HVP and brain structure was examined on a regional level by comparing bilateral MTL sub-volumes between HVP groups in two MANCOVAs. We hypothesized the MTL to exhibit an association to the HVP rather in its anterior than posterior parts because of the specificity of the AChA’s irrigation to the anterior part of the hippocampus. The first MANCOVA revealed significantly greater anterior MTL volumes (aHC, ERC, PRC) in augmented-supply individuals over individuals with a basic supply while accounting for age, sex and TIV (*F*(3, 34) = 3.613, *P* = 0.023). Neither a main effect of CSVD (*F* = 0.680, *P* = 0.564) nor an interaction between CSVD and the HVP (*F* = 0.689, *P* = 0.566) was found for anterior MTL volumes.

*Post* *hoc* univariate ANCOVAs for the different sub-regions of the anterior MTL identified the effect of the augmented supply to be especially driven by the ERC (*F*(1,36) = 11.259, *P* = 0.002, $\eta^{2}$ = 0.167, $\eta_{p}^{2}$ = 0.238). The aHC showed a strong trend (*F* = 4.030, *P* = 0.053, $\eta^{2}$ = 0.071, $\eta_{p}^{2}$ = 0.101), whereas there was no effect observed for the PRC (*F* = 0.777, *P* = 0.384, $\eta^{2}$ = 0.014, $\eta_{p}^{2}$ = 0.021). Permutation ANCOVAs obtained roughly the same results (see Supplementary Results).

**Figure 4:**
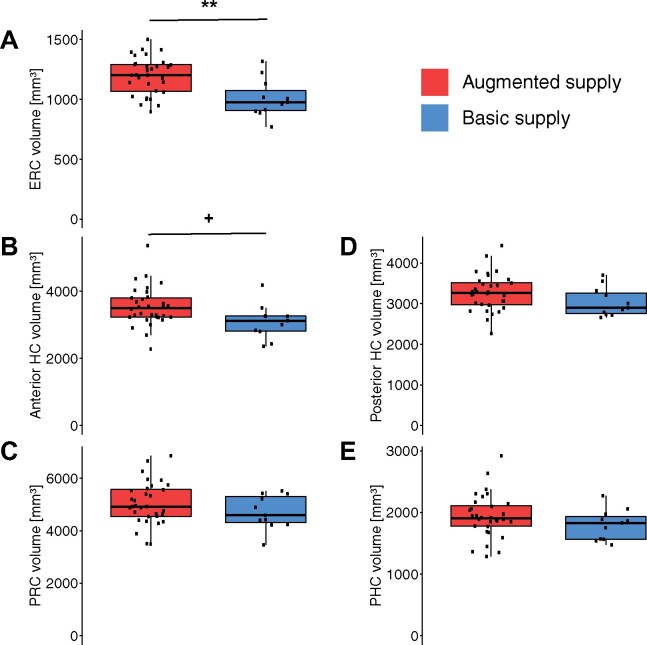
**Boxplot of bilateral MTL substructure volumes by subject-level HVP.** ROI volumes were obtained via segmentation of the desired substructures. ‘+’ denotes *P* *<* 0.1, ‘**’ indicates *P* *<* 0.01 from *post hoc* ANCOVAs. All *y*-axes start at 0 for better comparison.

In contrast to the anterior MTL volumes, the posterior MTL volumes (posterior hippocampus, parahippocampal cortex) were not associated with differences in the HVP as indicated by MANCOVA (*F*(2, 35) = 0.685, *P* = 0.511). Again, no main effect of CSVD (*F* = 0.531, *P* = 0.592) nor interaction (*F* = 0.915, *P* = 0.410) was present. Fig. 4 illustrates the differences in regional GM volume between single- and augmented-supply individuals for the selected MTL ROIs. In summary, individuals with an augmented HVP showed higher volumes in the aHC and ERC, but the lacking interaction between CSVD and HVP does not support the hypothesis of brain resilience on a structural level.

### Associations between HVP and GM volumes are more pronounced ipsilaterally than contralaterally

To take a differentiated look at the directness of the observed associations between GM volumes and the HVP, the analysis was extended from a subject to a hemisphere level. In an LME that accounted for the dependence of both hemispheres of the same individual, the contributions of the ipsilateral versus contralateral HVP were sought to be disentangled. If vascularization affects the structural integrity, one might speculate that nearby GM regions are affected stronger by a lack/surplus of supply when compared to more distant (contralateral) brain areas.

**Table 2:** Estimated effects of CSVD and the ipsi- and contralateral HVP on aHC and ERC volumes

	ΔaHC (mm^3^)	ΔERC (mm^3^)
CSVD	−88.79 [−331.21, 153.62]	−1.18 [−70.93, 68.57]
Dual_ipsi_	**147.37 [8.91, 285.83]**	**54.18 [11.42, 96.95]**
Dualcontra	84.54 [−53.92, 222.99]	**50.22 [7.46, 92.99**]
CSVD: Dual_ipsi_	−117.09 [−335.45, 101.26]	−23.42 [−92.55, 45.70]
CSVD: Dual_contra_	75.19 [−143.17, 293.54]	−20.47 [−89.59, 48.66]

GM volumes of other substructures were not examined, since there was no relation between the HVP and those in the subject-level model. The estimates come from an LME (separate for aHC and ERC) and can be interpreted as the average effect of the factor (e.g. presence of CSVD or an ipsi-/contralateral dual supply) on the given MTL volume in mm^3^. 95% confidence intervals are indicated in square brackets (*P*-values can be found in Supplementary Table 1). Significant results are shown in bold. ‘:’ symbolizes an interaction between two factors.

As Table 2 demonstrates, a dual supply in a given hemisphere was associated with greater aHC volumes in both the ipsilateral and contralateral hemisphere when compared to a single supply, on average amounting to 147.37 and 84.54 mm^3^, respectively. However, of all examined variables, only the ipsilateral HVP showed a significant effect, as indicated by the 95% confidence intervals, which do not contain 0. This means that the anterior part of the hippocampus is bigger in hemispheres with a dual supply compared to single-supply hemispheres.

In terms of ERC volumes, this significant association was not only observed for an ipsilateral but also a contralateral dual supply (Table 2), although the estimations differed slightly in their extent (54.18 mm^3^ for the ipsilateral compared to 50.22 mm^3^ for the contralateral hemisphere).

CSVD status and both interactions of CSVD with the HVPs were neither associated with regional differences in aHC nor ERC volumes. CSVD does not seem to be related to GM volume in MTL sub-regions specifically, confirming the findings of the previous (subject level) analysis. Also, in alignment with the subject-level analysis, this hemisphere-level analysis indicated a stronger relation of the HVP to ERC volume than to the aHC, as can be assessed by the (relatively) closer proximity of the confidence intervals to 0.

### Greater TGM volumes in augmented supply and controls

**Figure 5:**
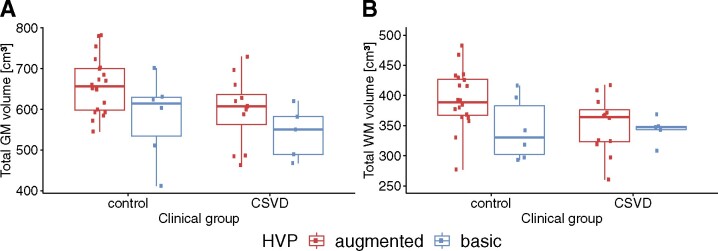
**Boxplot of TGM and WM volume by (subject level) HVP group and CSVD status.** For GM, a main effect of both CSVD status and the HVP was found. For WM, the main effect of the clinical group prevailed. No interaction effects were observed. Significance was tested in an ANCOVA.

Using ANCOVAs, we tested the hypotheses that TGM and total WM volumes differ based on the HVP. In terms of TGM volumes, the analysis revealed a strong association between CSVD status and GM volume (*F*(1,36) = 8.0994, *P* = 0.007, $\eta^{2}$ = 0.086, $\eta_{p}^{2}$ = 0.184), i.e. CSVD patients had significantly reduced GM volume relative to controls (see Fig. 5A). The relation between the HVP and TGM volume was also significant (*F* = 6.9915, *P* = 0.012, $\eta^{2}$ = 0.074, $\eta_{p}^{2}$ = 0.163). In this case, augmented-supply individuals had significantly greater TGM volume when compared to basic-supply individuals. When accounting for the anterior MTL volumes by subtracting them from the TGM volumes, the results remained unchanged (CSVD: *P* = 0.007; HVP: *P* = 0.013). To ensure robustness, a permutation test for ANCOVA was executed that presented a weaker association with CSVD (*P* = 0.024), but similar results for the HVP (see Supplementary Results). A voxel-based morphometry (VBM) analysis indicated that regions with greater local GM volumes in the augmented-supply groups were located in the temporal lobe or in close proximity to it (see Supplementary Results). An interaction between HVP and CSVD status was not present (*F* = 0.1851, *P* = 0.670, $\eta^{2}$ = 0.002, $\eta_{p}^{2}$ = 0.005).

ANCOVA with permutation revealed no significant associations between total WM volume (boxplots for HVP-CSVD pairs shown in Fig. 5B) and the HVP (*F* = 2.3663, *P* = 0.134, $\eta^{2}$ = 0.037, $\eta_{p}^{2}$ = 0.062) or CSVD (*F* = 2.4419, *P* = 0.131, $\eta^{2}$ = 0.038, $\eta_{p}^{2}$ = 0.064). An interaction was also not observed (*F* = 1.3161, *P* = 0.254, $\eta^{2}$ = 0.020, $\eta_{p}^{2}$ = 0.035).

In summary, while greater TGM volumes were seen in the presence of an augmented vascular supply across all individuals, our models do not reveal evidence for structural brain resilience with respect to CSVD.

### TGM volume is a mediator between the HVP and cognition

**Figure 6:**
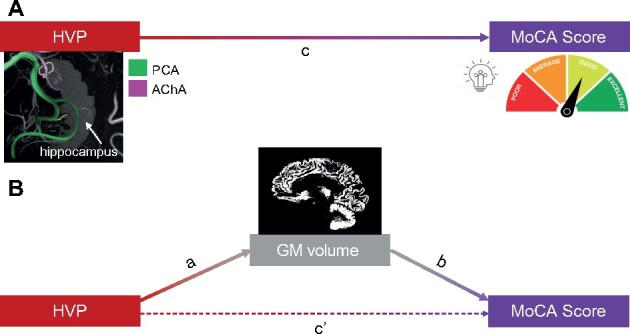
**Mediation analysis. Mediation scheme after Preacher and Hayes^38^ shown for the relation between HVP, TGM volume and MoCA Score.** **A**) The total effect of the subject-level HVP on the MoCA score. **B**) The average direct effect is represented by cʹ. The average causal mediation effect is represented by a*b. Together, average direct effect and average causal mediation effect amount to the total effect (c = cʹ + ab).

The relation between the (subject level) HVP and global cognition as observed by Perosa et al.^13^ in combination with the relation between HVP and TGM volume gave rise to the hypothesis that the cognitive performance differences might be grounded in the global structural differences (schematically shown in Fig. 6). A mediation analysis was employed to support the hypothesis that the differences in global cognition between augmented- and basic-supply individuals are caused by the TGM differences. The mediation analysis confirmed a significant total effect (path c in Fig. 6A) of the HVP on global cognition represented by the MoCA score (*P* = 0.017). The average direct effect (path cʹ in Fig. 6B) was not statistically significant (*P* = 0.130). In contrast, the average causal mediation effect (path a*b in Fig. 5B), which is the difference between the total and average direct effect (a*b = c—cʹ), was statistically significant (*P* = 0.050). These findings are compatible with the hypothesis that better global cognition in augmented-supply individuals is caused by HVP-related differences in TGM volume.

To rule out the possibility that these effects are only driven by higher GM volumes in the MTL, relationships between HVP, MoCA score and anterior MTL volumes were investigated additionally (see Table 3). As previously shown, an augmented supply was associated with bigger anterior hippocampal and entorhinal GM volumes. However, greater GM volumes in the anterior MTL were not related to better performance on the MoCA score. Thus, they cannot mediate the effect of the HVP on global cognition.

**Table 3:** *P-*values from ANCOVAs between different variables and cognition/memory scores

	MoCA score	CVLT composite score
HVP	**0.017**	**0.039**
TGM vol	**0.011**	0.449
ERC vol	0.440	0.254
aHC vol	0.660	0.349

These relationships were tested as a prerequisite for the mediation analysis. Each combination of row and column presents a distinct model. Significant results are printed in bold.

Moreover, Perosa et al.^13^ established a connection between the HVP and verbal memory scores. Since memory is a key function of the hippocampus and MTL, we hypothesized that aHC or ERC volumes mediate the relationship between the HVP and memory functions. A composite CVLT measure served as a combined memory score, which was positively correlated with the HVP (see Table 3). Yet, neither the examined MTL volumes nor TGM volume were significantly related to the composite CVLT score.

In summary, the mediation analysis indicates that greater TGM volumes are responsible for better cognition in the augmented-supply group, not greater anterior MTL volumes. In contrast, better (verbal) memory in individuals with an augmented supply could be explained neither by global differences in GM nor anterior MTL volumes alone.

### No evidence for resistance nor resilience against CSVD

We hypothesized that the HVP confers resilience against CSVD on a structural level, i.e. that individuals with an augmented supply show higher than expected TGM volume given their pathology. This would be represented by an interaction of the presence of CSVD with the HVP. However, no such interaction was observed in any of the models, as stated in the previous results sections.

Assuming that pathological markers of CSVD are similarly distributed among the population (accounting for age, sex and TIV), we further hypothesized that resistance against CSVD would imply lower levels of pathological markers in individuals with an augmented supply. Resistance was examined at multiple levels, namely in the local context of the hippocampus and the global context of the whole brain. The number of hippocampal microinfarcts as a local pathology measure did not reveal any differences between the two HVP groups (*F*(1,38) = 0.9141, *P* = 0.345).

**Figure 7:**
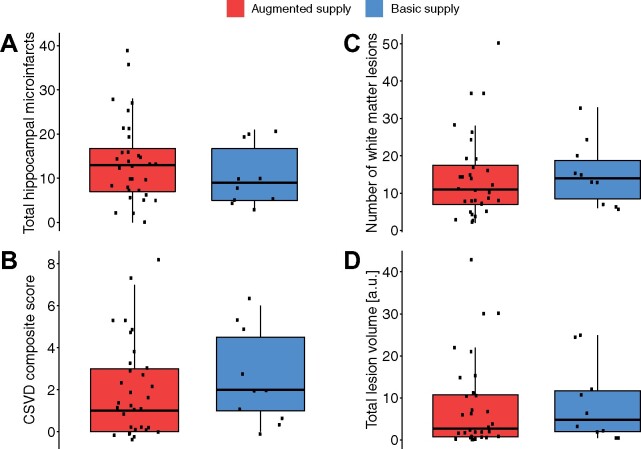
**Boxplots of different CSVD measures by subject-level HVP.** Included are both local (**A**) and global measures (**B–D**) of CSVD. ANCOVAs revealed no differences between the augmented- and basic-supply group. Panels (**C)** and (**D**) were obtained by the LPA algorithm of the LST toolbox.

To allow a better evaluation of resistance in regard to the whole brain, CSVD was not considered solely as present or absent, but examined in a more quantitative way by converting it to a novel semi-quantitative score in the previously described manner. However, an augmented supply was not related to a lower whole-brain CSVD score either (*F*(1,38) = 0.3263, *P* = 0.571). A MANCOVA on total lesion volume and the number of lesions as identified by WMH showed the same result (*F*(2,35) = 0.2123, *P* = 0.810). Boxplots of the measures for the different groups are shown in Fig. 7. The lack of differences in pathological measures of CSVD between the HVP groups represents a lack of evidence for resistance against CSVD in both a local and a global brain context.

Using Fisher’s exact test, we further found no statistical difference in clinical group contingency (*P* = 0.25).

## Discussion

Recent advances in ultra-high-resolution ToF magnetic resonance angiography enabled us to classify the HVP of 85 brain hemispheres *in vivo*. We examined cross-sectional associations of the HVP with brain structure on a scope far beyond that of previous studies, considering the laterality and vicinity (regional versus global) of those associations. We further investigated the structural underpinnings that might underlie the positive association between an augmented hippocampal vascular supply and cognition. We also examined whether an augmented vascularization was related to reduced vascular pathology burden (resistance against pathology) or preserved GM integrity in the face of vascular burden (resilience to pathology), thus making the connection of established and novel structural markers to these two core concepts in ageing and disease.

A comparison between individuals with a basic supply and individuals with an augmented supply, i.e. at least one hemisphere in which anterior circulation directly contributes to hippocampal blood supply via the AChA, revealed bigger anterior but not posterior MTL volumes. This matches the specificity of AChA perfusion to the hippocampal head and the anterior part of the parahippocampal gyrus.^5^^,^^41^ Such a specific benefit in anterior integrity could have implications for memory function, as a functional gradient along the longitudinal axis of the hippocampus has been proposed.^42^ Notably, the aHC shows an age-related decrease in functional connectivity that may contribute significantly to memory decline in older adults.^43^ The hypothesis that brain structures benefit more from a direct effect of vascularization than indirect is supported by our observation that the volume gains for aHC and ERC were stronger for ipsilateral than contralateral dual supply.

There are likely several mechanisms through which an augmented supply and GM volumes are related to each other. First, dual vascularization could be associated with better blood supply with nutrients and oxygen as well as better clearance of harmful substances and metabolic end-products. This may promote demand-driven plasticity, including synaptogenesis and neurogenesis, which is known to be coupled with angiogenesis (for a review see Duzel et al.^44^). Hippocampal neuronal plasticity, in turn, is known to be associated with the plasticity of intra- and extra-hippocampal connectivity. In consequence, a local increase of hippocampal plasticity-potential by improved vascularization may have spill-over effects to hippocampal networks, which could also extend to contralateral homotopic regions, as previously shown and observed for the ERC in this study.^45^ While the ERC has been shown to be irrigated by arteries originating from the AChA,^46^ it remains unclear why the hypothesized effect of AChA supply is stronger for the ERC than the hippocampus.

Strikingly, an augmented supply of the hippocampal region was also associated with greater GM volume beyond the MTL. Our data do not provide any mechanistic explanation for these remote, global associations. One interpretation is that cerebral vascularization in other areas co-varies with hippocampal vascularization, e.g. in the anterior circulation. As such, the HVP might represent a proxy for blood supply in multiple brain regions. Another interpretation is that an increased potential for hippocampal plasticity has a long term, maybe even developmental, impact on cortical and sub-cortical networks. Such widespread structural covariance has been reported in chronic conditions affecting the hippocampus such as epilepsy due to medial temporal sclerosis.^47^^,^^48^ Of note, greater anterior MTL volumes were not able to numerically account for the observed positive association between a dual supply and greater whole-brain volume. Our VBM analysis revealed volumetric gains for augmented over basic supply in extra-hippocampal regions, especially those in relative proximity to the MTL.

Our hypothesis that whole-brain differences in GM are responsible for the HVP-related better global cognition was supported by the significant mediation effect of TGM. Yet, there might be more direct mediators, e.g. blood perfusion or differences in whole-brain vascularization (co-varying with the HVP) that were not measured here. Surprisingly, the composite CVLT score which was used as a proxy for memory was not significantly correlated with anterior MTL volumes. This indicates that the positive association of the HVP with (verbal) memory is grounded in more widespread structural covariance in the memory network, perfusion or patterns of neural activity. Of note, no non-verbal memory modalities were acquired in the current study to assess memory in a broader way.

In patients with CSVD, TGM volumes were significantly smaller compared to controls. This is in correspondence with other studies that reported neurodegeneration in sporadic CSVD.^49^^,^^50^ There were no volumetric differences between CSVD patients and controls in MTL substructures. This is in line with the lack of specificity of CSVD to certain brain regions.^51^

Our data did not reveal any cross-sectional evidence to support the hypothesis that the HVP conveys resilience against vascular pathology, since there was no interaction effect between CSVD and the HVP on GM volumes, meaning that the HVP was not associated with greater than expected brain structures. Certainly, resilience ultimately needs to be assessed in a longitudinal design that allows for HVP-dependent trajectories. Furthermore, our analyses did not indicate that an augmented supply protects individuals from vascular pathology. Yet, CSVD is a whole-brain disease with a very complex aetiology (e.g. age, vascular risk, genetics, lifestyle), which makes it unlikely to observe HVP-conveyed resistance against CSVD on the whole-brain level. However, there was also no evidence for hippocampus-specific resistance against vascular pathology when examining the sole measure of hippocampal microinfarcts.

Notably, the current investigations were restricted to downstream MRI measures of CSVD and do not provide insight about other diseases like Alzheimer’s disease, in which resistance and resilience conferred by the HVP are imaginable and could be assessed in relation to tau or Aβ load. Here, our finding of a potential structural protection of the ERC by an augmented vascular supply is of particular importance, since the ERC is highly vulnerable to tau pathology,^52^^,^^53^ and early atrophy in ageing and Alzheimer’s disease.^54^

While the HVP might also play a distinct role for hippocampal subfield integrity, we believe that subfields could not be faithfully segmented because the stratum radiatum lacunosum moleculare as a necessary landmark is not clearly identifiable in our images due to the lack of a high-resolution T_2_ hippocampal scan (see Wisse et al.^55^ regarding limitations for segmentation of hippocampal subfields). As the utilized classification is agnostic to the location of small hippocampal arteries, which are beyond the resolution limit of the ToF, it is hard to make predictions about the potential impact of the HVP on hippocampal subfield integrity. However, the subiculum or CA1 might be more likely to benefit from contribution by the AChA, as they extend more anterior compared to other subfields.^56^ Moreover, previous studies reported a selective vulnerability of the CA1 region to conditions like hypoxia, ischaemia and vascular pathology,^57–59^ providing further indications for an important role of the HVP for CA1, which should be addressed in future studies.

Despite the interesting and extensive findings in this study, one has to be aware of certain limitations. Since the data are purely cross-sectional, it does not allow any inference about time courses. Although our hypotheses and models imply a clear directionality (HVP affects structure affects cognition), we cannot exclude reverse causality, meaning that a bigger brain structure simply requires a better blood supply. Given the limited statistical power and unbalanced design (32 augmented-supply versus 11 basic-supply subjects), one should be careful about over-interpretations.

Moreover, it is conceivable that there was a small degree of under-classification of dual-supply hemispheres due to overlooked uncal branches beyond the detection threshold, as suggested by Wiesmann and de Leeuw.^60^ We note, however, that the proportion of dual-supply hemispheres does not differ significantly from the proportion observed by Erdem et al.^5^ (Fisher’s exact test, *P* = 0.53).

One also has to note the qualitative character of this study. The HVP is not a quantitative measure of hippocampal blood supply and it has yet to be examined how the HVP relates to hippocampal perfusion. From a qualitative point of view, it might be interesting to better understand the contribution of the anterior and posterior circulation to hippocampal vascularization and its role for downstream effects by considering variations in the Circle of Willis. Relevant variants in that context could be a foetal or hypoplastic PCA as well as hypoplasticity or absence of the posterior communicating artery, as also suggested by Gutierrez.^61^ Nonetheless, the strength of the presented classification is its simplicity in the light of its apparent relevance, granting it a translational character.

The finding that augmented-supply individuals have more preserved regional and TGM volumes than age-matched basic-supply individuals is a first indicator that the HVP might contribute to brain reserve or brain maintenance (as defined by Stern et al.^62^) in the context of ageing. Notably, this evidence requires confirmation from longitudinal studies. If a single hippocampal supply represents a predictor for cognitive decline later in life, this could have implications for risk modification and preventive therapies, as these individuals might be more vulnerable to develop dementia. Finally, the pattern of hippocampal vascularization might be an important modifier of hippocampal plasticity. We have shown that exercise-related vascular plasticity is highly variable among older adults,^63^ suggesting that other factors, such as the vascularization patterns in the MTL might modify exercise-related benefits.

To summarize, future studies should examine the relationship between the HVP and quantitative perfusion measures in the hippocampus and MTL. Moreover, investigating the relation between the HVP and brain structure in young adults would help to understand its role in brain development. Longitudinal data will be required to distinguish higher baseline levels prior to the onset of atrophy from slower rates of structural (and cognitive) decline. Furthermore, investigation of the implications in resistance and resilience against Alzheimer’s disease appears very worthwhile given the overlap of the area where tau first accumulates (see Braak and Braak^52^) with the area that seems to benefit the most from the HVP.

## Supplementary material

Supplementary material is available at *Brain Communications* online.

## Funding

This project was funded by the Deutsche Forschungsgemeinschaft (DFG, German Research Foundation) - Project-ID 425899996 - SFB 1436. H.M. is supported by DFG - grant number: MA 9235/1-1.

## Competing interests

The authors report no competing interests.

Box 1 Definitions^12^

**Resistance**:   Protection against pathology, i.e. absence or lower than expected levels of pathology.

**Resilience**:   Remaining normal despite considerable pathology, i.e. (i) higher than expected brain structure/function given observed pathology or (ii) more than expected pathology given observed brain structure/function.

## Supplementary Material

fcab127_Supplementary_Data

## Abbreviations

AChA =: anterior choroidal artery
aHC =: anterior hippocampus
ASHS =: Automatic Segmentation of Hippocampal Subfields
CAA =: cerebral amyloid angiopathy
CDR =: Clinical Dementia Rating
CMB =: cerebral microbleeds
CSVD =: cerebral small vessel disease
CVLT =: California Verbal Learning Test
ERC =: entorhinal cortex
GM =: grey matter
HVP =: hippocampal vascularization pattern
LME =: linear mixed effect
MMSE =: Mini-Mental State Examination
MoCA =: Montreal Cognitive Assessment
MTL =: medial temporal lobe
PCA =: posterior cerebral artery
PRC =: perirhinal cortex
PVS =: perivascular spaces
ROI =: region of interest
TGM =: total grey matter
TIV =: total intracranial volume
ToF =: time-of-flight
VBM =: voxel-based morphometry
WM =: white matter
WMH =: white matter hyperintensities
