# Supplementary material for: Hippocampal vascularization patterns exert local and distant effects on brain structure but not vascular pathology in old age
Source: Brain Commun. 2021 Jun 4;3(3):fcab127. doi: 10.1093/braincomms/fcab127 (PMC8249103; doi:10.1093/braincomms/fcab127)
Supplement: fcab127_Supplementary_Data [file fcab127_supplementary_data.pdf]

Supplementary

Supplementary Methods

Statistical analysis

In order to make inference on the LME results, confidence intervals were calculated. Confidence intervals are already sufficient to guide inference because they provide information direction and strength of the effect as well as about statistical significance. Hence, we want to emphasize that there is no absolute need for p values. Nevertheless, we calculated p values in order to supply some complementary information, i.e. the probability to observe a distribution as extreme as the one observed under the assumption of the null hypothesis that the factors are not associated with GM volumes of the aHC/ERC. Thus, LRTs between the full model and a full model minus the variable in question were additionally performed to obtain a p value for each respective dependent variable, i.e. five LRTs were performed per independent variable.

VBM

In order to assess the local effects of the HVP on brain structure on a voxel-by-voxel basis in an unbiased way within the whole brain, a VBM analysis was performed on the 7T T1 images with SPM12 after preprocessing with the CAT12 toolbox (version 12.6)[64]. Preprocessing included segmentation, normalization and smoothing steps. The SPM segmentation routine, which performs segmentation, bias correction and spatial normalization in the same model, was performed with the following parameters deviating from their default values: biasstr = 1 (strong SPM inhomogeneity correction), accstr = 1 (high SPM processing accuracy), vox = 1 (1 mm voxel size for normalized images). An 8 mm Gaussian kernel was used for smoothing.

The subsequent statistical analysis in SPM employed a two-sample t-test to identify regions with different local GM volumes between the basic- and augmented-supply group. On top of the the covariates previously included in the (M)ANCOVAs, the presence/absence of CSVD was included as an additional covariate. Clusters of significant voxels were examined at an uncorrected threshold of $p < 0.005$ at the voxel-level, with different minimal cluster sizes and different corrections at the cluster-level (uncorrected or corrected for false discovery rate (FDR)). To get an additional impression of the location of those clusters, the Neuromorphometrics Atlas in SPM was used to roughly label them as anatomical structures.

Supplementary Results

TGM volumes

Permutation ANCOVAs on GM volumes of anterior MTL substructures obtained the following results, which were very similar to the ANCOVAs without permutation testing: The ERC showed a quite significant effect ($F(1,36) = 10.221$, $p = 0.0036$, $\eta^2 = 0.156$, $\eta_p^2 = 0.221$) and the anterior hippocampus showed a strong trend ($F = 3.943$, $p = 0.053$, $\eta^2 = 0.070$, $\eta_p^2 = 0.099$), whereas no relation was observed between the HVP and PRC ($F = 0.567$, $p = 0.456$, $\eta^2 = 0.010$, $\eta_p^2 = 0.016$).

When examining the relation of the HVP and CSVD status with TGM, permutation testing produced similar results as the regular ANCOVA for HVP ($F(1,36) = 6.575$, $p = 0.0134$, $\eta^2 = 0.072$, $\eta_p^2 = 0.154$), but a weaker association of CSVD with TGM ($F = 5.566$, $p = 0.0225$, $\eta^2 = 0.061$, $\eta_p^2 = 0.134$). The interaction effect was not significant ($F = 0.185$, $p = 0.665$, $\eta^2 = 0.002$, $\eta_p^2 = 0.005$).

VBM analysis

After identification of differences in TGM due to the HVP, an exploratory VBM analysis was utilized to shed some additional light on local drivers of those overall differences. Supplementary Figure 1 displays regions with higher GM volume in subjects with augmented than basic supply (p_{voxel} (uncorrected) < 0.005; $k > 1000$). Eight regions can be identified this way (see also Supplementary Figure 2). Notably, the bottom cluster in Supplementary Figure 1E seems to be located around the anterior hippocampus ($p_{cluster} = 0.094$ after FDR-correction). Applying an FDR-correction at the cluster-level, four clusters remain significant ($p < 0.05$) which appear to be located in or close to the temporal lobe. According to the Neuromorphometrics Atlas, these cover the right middle temporal gyrus (Supplementary Figure 1A), the left supramarginal gyrus or postcentral gyrus (Supplementary Figure 1C and D) and the left and right transverse temporal gyrus (Supplementary Figure 1E).

Supplementary Figures and Tables

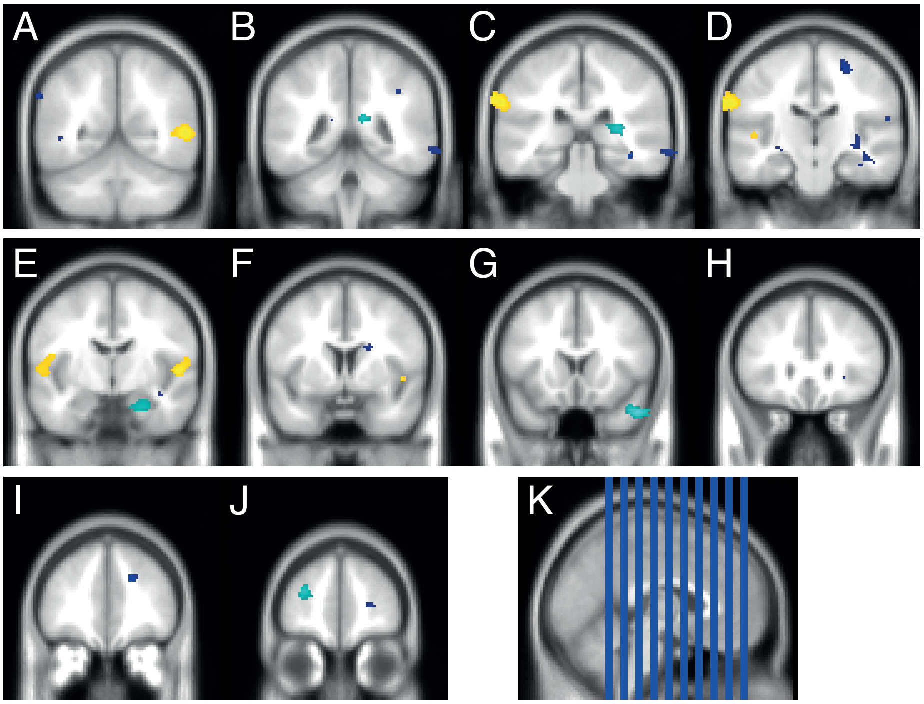

Supplementary Figure 1: Results of the exploratory VBM. (A-J) Regions where subjects with an augmented vascular supply of the hippocampus exhibit greater GM volume than subjects with a basic supply ($p_{voxel} < 0.005$; uncorrected). Blue color indicates clusters with at least 20 voxels, cyan shows clusters which contain at least 1000 voxels, yellow represents clusters with $p < 0.05$ after FDR-cluster correction. (K) Sagittal location of coronal slices A-J.

Statistics: ***p-values adjusted for search volume***

set-level		cluster-level				peak-level							
p	c	$p_{FWE-corr}$	$q_{FDR-corr}$	k_E	p_{uncorr}	$p_{FWE-corr}$	$q_{FDR-corr}$	T	$(Z_{\equiv})$	p_{uncorr}	mm	mm	mm
**0.000**	**8**	**0.117**	**0.051**	**1530**	**0.001**	**0.914**	**1.000**	**4.62**	**4.08**	**0.000**	**54**	**22**	**-34**
						0.928	1.000	4.60	4.06	0.000	50	16	-30
						1.000	1.000	3.01	2.83	0.002	58	16	-28
		**0.007**	**0.007**	**2591**	**0.000**	**0.919**	**1.000**	**4.62**	**4.08**	**0.000**	**66**	**-66**	**4**
						0.998	1.000	4.23	3.79	0.000	56	-58	4
						1.000	1.000	3.00	2.82	0.002	42	-54	6
		**0.152**	**0.056**	**1436**	**0.001**	**0.946**	**1.000**	**4.55**	**4.03**	**0.000**	**-28**	**46**	**12**
		**0.000**	**0.001**	**3942**	**0.000**	**0.976**	**1.000**	**4.45**	**3.96**	**0.000**	**-70**	**-20**	**30**
						0.999	1.000	4.20	3.77	0.000	-64	-28	30
						1.000	1.000	4.01	3.63	0.000	-70	-32	34
		**0.205**	**0.067**	**1328**	**0.002**	**1.000**	**1.000**	**4.14**	**3.73**	**0.000**	**12**	**-40**	**16**
						1.000	1.000	3.86	3.52	0.000	30	-32	8
						1.000	1.000	3.40	3.15	0.001	24	-40	12
		**0.017**	**0.012**	**2237**	**0.000**	**1.000**	**1.000**	**4.00**	**3.62**	**0.000**	**54**	**-14**	**4**
						1.000	1.000	3.35	3.11	0.001	70	-0	10
						1.000	1.000	3.18	2.97	0.001	46	2	-4
		**0.305**	**0.094**	**1183**	**0.003**	**1.000**	**1.000**	**3.46**	**3.20**	**0.001**	**26**	**-10**	**-26**
		**0.069**	**0.037**	**1716**	**0.001**	**1.000**	**1.000**	**3.36**	**3.12**	**0.001**	**-48**	**-10**	**14**
						1.000	1.000	3.34	3.10	0.001	-52	-12	4
						1.000	1.000	3.19	2.98	0.001	-46	-20	2

Supplementary Figure 2: Clusters with at least 1000 voxels with $p_{uncorrected}$ as identified in the VBM analysis. FWE = family-wise error, FDR = false discovery rate, k = cluster size.

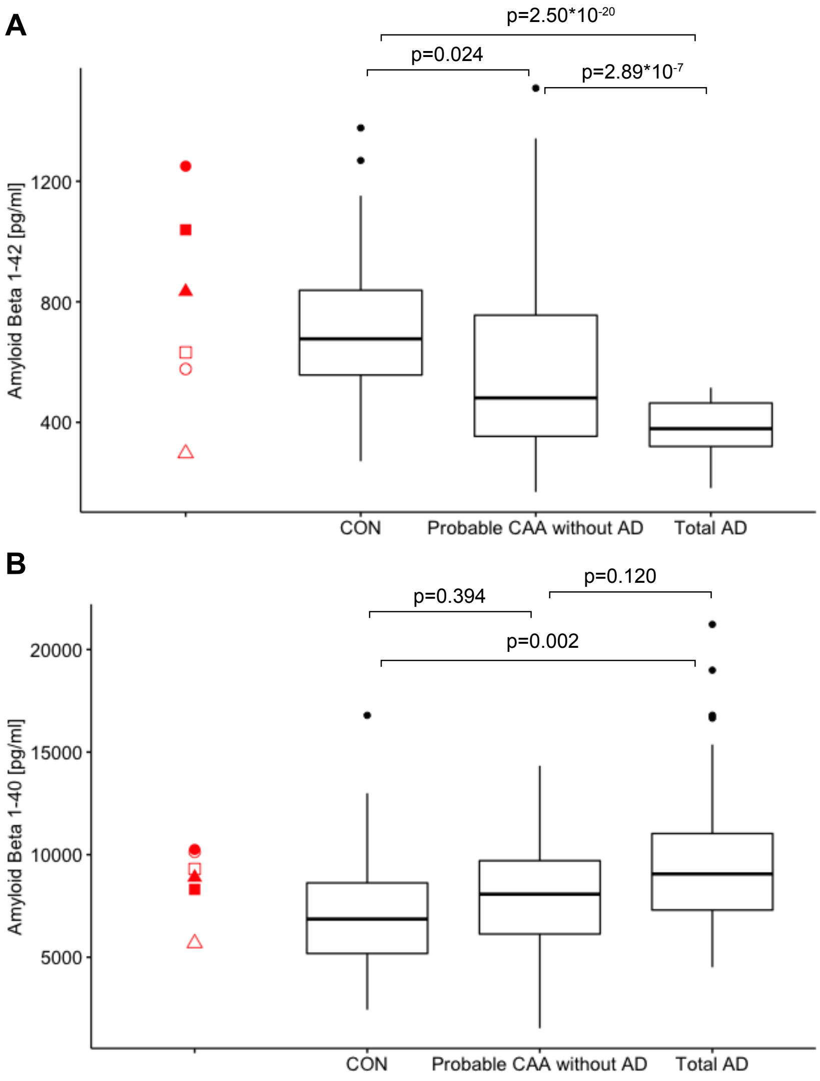

Supplementary Figure 3: Boxplots of CSF (A) Aβ_{1-42} and (B) Aβ_{1-40} concentrations across different clinical groups of a large cohort of older adults. Probable CAA diagnosis was based on the modified Boston criteria (n = 60).[17] CON includes HA patients, whose diagnosis was based on the existence of deep microhemorrhages, and controls, who did not reveal any CSVD according to STRIVE and were negative for AD based on the combination of Aβ_{1-42} and p-tau in CSF (n = 48).[11] AD diagnosis was based on the NINCDS-ADRDA criteria and the ATN classification with concurrent Aβ_{1-42} and a p-tau positivity (n = 91).[19] The red symbols distinctly represent the six CAA patients in the current study. Group comparisons were conducted using an ANCOVA adjusting for age and sex.

Supplementary Table 1: Estimated effects of CSVD and the ipsi- and contralateral HVP on aHC and ERC volumes as shown in Table 2. p values from likelihood ratio tests are indicated in round brackets. Significant results shown in bold. ':' symbolises an interaction between two factors.

	ΔaHC [mm^3]	ΔERC [mm^3]
CSVD	-88.79 (0.464)	-1.18 (0.973)
$Dual_{ipsi}$	**147.37 (0.037)**	**54.18 (0.014)**
$Dual_{contra}$	84.54 (0.227)	**50.22 (0.022)**
CSVD:$Dual_{ipsi}$	-117.09 (0.288)	-23.42 (0.502)
CSVD:$Dual_{contra}$	75.19 (0.494)	-20.47 (0.557)
